# Can Migration Health Assessments Become a Mechanism for Global Public Health Good?

**DOI:** 10.3390/ijerph111009954

**Published:** 2014-09-26

**Authors:** Kolitha Wickramage, Davide Mosca

**Affiliations:** 1 International Organization for Migration, Country Office Sri Lanka, Colombo 3, Sri Lanka; 2 Migration Health Division, the International Organization for Migration, Route des Morillons 17, Geneva, 1211 CH, Switzerland; E-Mail: dmosca@iom.int

**Keywords:** migrant health, health assessment, tuberculosis screening, migrant rights

## Abstract

Migrant health assessments (HAs) consist of a medical examination to assess a migrant’s health status and to provide medical clearance for work or residency based on conditions defined by the destination country and/or employer. We argue that better linkages between health systems and migrant HA processors at the country level are needed to shift these from being limited as an instrument of determining non-admissibility for purposes of visa issuance, to a process that may enhance public health. The importance of providing appropriate care and follow-up of migrants who “fail” their HA and the need for global efforts to enable data-collection and research on HAs are also highlighted.

## 1. Introduction

Today, more people are “on the move” than at any other time in recorded history [1]. Although there are many categories of migrants, the scope of this paper focuses on international migrants, defined by the United Nations as “persons born in a country other than that in which they reside in” [2]. There are an estimated 232 million international migrants, which, if these were their own country, would be the sixth largest nation in the world [1]. International migration forms a key pillar in globalization. Remittances from migrant workers account for almost 90 percent of the total stock of international migrants [3], making significant contributions to economic development and foreign exchange reserves [4]. Remittances also contribute to the achievement of the Millennium Development Goals by reducing poverty through the provision of income at the household level, which is spent on food, shelter, education and health.

## 2. Migration Health Assessments (HAs)

**Figure 1:**
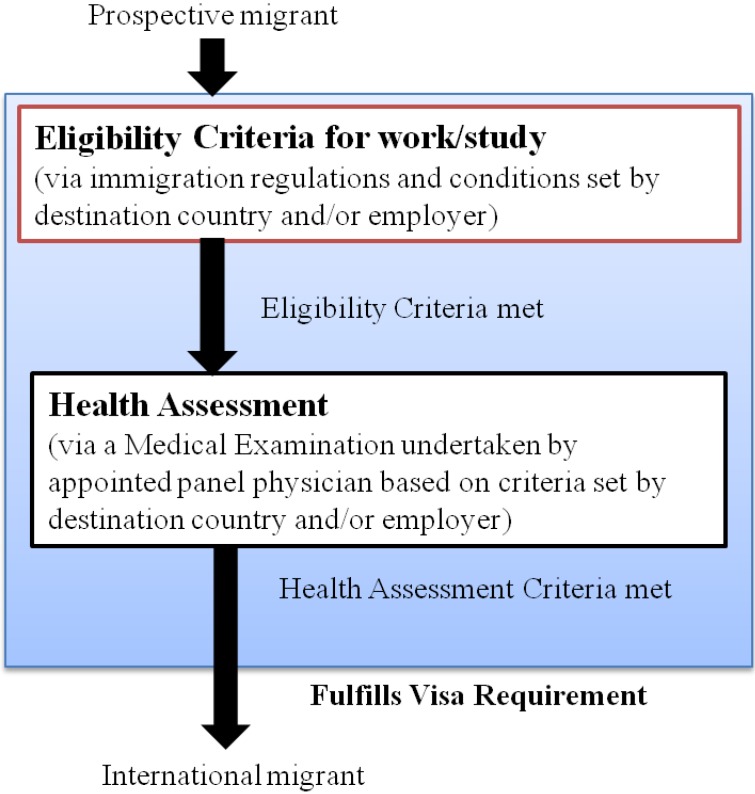
A basic model showing how the health assessment (HA) is a linked migration process.

Health assessments (HAs) form an integral part of many immigration and labor migration programs worldwide. At its core, the HA is essentially a medical examination, usually conducted by a registered medical practitioner (or “panel physician”) based on a criteria set by the country or employer of their intended destination (‘destination country’). They are regulated through the immigration processes and labor laws of destination countries as part of a person’s visa requirement (Figure 1). The origin of pre-departure HAs may be traced to their introduction at the end of the First World War, when major immigrant-receiving nations established off-shore medical screening programs for prospective migrants [5]. Migrants intending to work, study or seek residency in a country on a permanent basis or for a temporary period of time are required to undertake the medical examination. HAs are also undertaken for refugees and humanitarian entrants as part of resettlement programs and for irregular/undocumented migrants usually at post-arrival immigration holding/detention centers [6]. The focus of this paper considers only those undertaking HA as part of formal migrant programs and, thus, excludes this latter group.

## 3. Challenge of Estimating the Magnitude of HAs World-Wide

There are currently no global estimates on the total number of international migrants that undertake HAs, the sites and countries performing the screening, rates of disease detected and treatment outcomes. Baseline estimates of international migrants disaggregated to categories, such as labor migrants, students and humanitarian entrants, are also difficult to obtain at the global level [1]. The lack of data and limited evidence makes it difficult to quantify the magnitude of those undertaking HAs globally.

Data from seven government registries of known international migrant worker populations from the Asian region may provide an insight into the volume and dynamics of HAs for international labor migrants (Table 1). All migrant workers intending to work in Countries of the Gulf Cooperation Council (GCC), such as Qatar and Saudi Arabia, are required to undertake an HA at designated GCC clinics/panels in their countries of origin, with most requiring a follow-up examination after arrival [7].

**Table 1:** Outflow of migrant workers from selected Asian countries in 2012.

Country	Population (millions) in 2013 ^1^	Poverty Rate ^2^	Estimated stock of emigrants in 2013 ^3^	Registered Labor Migrants to GCC ^4^ nations in 2012 ^5^	Remittances (USD Bn) in 2012 (% of GDP) ^6^
**Bangladesh**	156.60	31.5	5,635,489	457,590	14.12 (12.2%)
**India**	1252	29.80	6,845,565	722,139	68.82 (3.7%)
**Nepal**	27.80	26.6	591,199	1,611,085	4.793 (24.7%)
**Pakistan**	182.10	22.3	3,557,855	628,452	14.01 (6.1%)
**Sri Lanka**	20.48	8.9	829,818	247,431	6.01 (10.1%)
**Indonesia**	249.90	12.5	1,336,688	603,159	7.212 (0.8%)
**Philippines**	98.39	26.5	2,380,669	791,765	24.64 (9.8%)
**Total**			**21,177,283**	**5,061,621**	**139.605 Bn**

Notes: **^1,2^** World Bank (2013) Country Data Base. **^3^** United Nations (2013) Department of Economic and Social Affairs; **^4^** Gulf Cooperation Council (GCC) includes the following countries: Bahrain, Kuwait, Oman, Qatar, Saudi Arabia, United Arab Emirates; **^5^** Figures are from government statistical sources from each individual country; **^6^** World Bank (2014) Annual Remittances Data (April 2014).

In 2012 alone, over five million international migrant workers had successfully “passed” the HA requirement to enable them entry for work (Table 1). Remittances from such migrant workers significantly contributed to the GDP of these nations. Despite the large volume of tests conducted, the HA case-load represented only 24% of the total estimated stock of international migrants in these seven countries. The data also underestimates the actual numbers of those undertaking the HA. For instance, migrants who are seeking to gain residency to non-GCC countries in Europe or America and as international students are not included. More importantly, the actual number who undertook the medical examination, those made non-admissible and the results of such tests are not published. There are no requirements or indeed global efforts for those undertaking HAs to publish such data. Since the data only includes those migrants that had formally registered with foreign employment agencies, it excludes those who travel via undocumented or “irregular” migration routes [8].

Developed nations with extensive immigration recruitment programs, such as Australia, Canada and the USA, also utilize HA models that are conducted at the migrant’s country of origin [9,10]. Although data on exact numbers of HAs undertaken each year are not published, it is estimated that, the collectively, five countries of the USA, Canada, Australia, U.K. and New Zealand undertake approximately two million immigration medicals annually [11]. The British Colombia Centre for Disease Control estimates that approximately 450,000 immigration medication examinations are completed annually, 350,000, which are undertaken through overseas panels and 95,000 undertaken in Canada [12].

## 4. Diversity in HA Models and Diseases Screened

Countries maintain their sovereignty in deciding who to admit in their country and the rules regarding non-admissibility. The purpose and rationale for conducting HAs for migrants are usually articulated in documents describing visa rules/regulations. HAs are very common in sectors that largely recruit migrant laborers, such as domestic maids and construction workers [13]. A number of countries require migrant workers to undertake an on-arrival medical exam and follow-up exams at regular intervals as a condition for maintaining their work and residency permit [7,14,15,16].

HAs are usually conducted as a measure to limit or prevent transmission of diseases of public health importance to their host populations; and to avert potential costs and burden on local health systems, especially for the treatment of chronic disease conditions [17]. The conditions examined are stipulated within screening protocols and technical instruction notes developed by destination countries and/or employers [18]. The concept of ‘normality’ in health status determination is dictated by the admissibility criteria and where the threshold for non-admissibility is placed. A study by Alvarez (2011) highlighted the diverse range of HA models across sixteen countries that differed across diagnostic protocols used, for example, to screen for tuberculosis (TB), the site of testing and the category of migrants to be tested [19].

The diseases detected are also difficult to generalize and compare, since they depend upon the definition of “admissibility” of the concerned immigration countries. In the case of tuberculosis (TB), for example, it depends on whether the screening is done to detect active, infectious disease, and therefore, the screening protocol is based on clinical, radiological and laboratory findings or latent TB, largely based on the tuberculin skin test.

## 5. Non-Admissibility: Those Who “Fail”

Public health consequences on those failing the HA are difficult to assess, considering most authorities seldom publish data on potential migrants who have undergone screening, the types of disease conditions and follow-up or referral outcomes. A paper by Elwood (2009) estimated that of the 450,000 immigration medication examinations that are completed annually by Citizenship and Immigration Canada (CIC), 55% arrived in Canada, of whom 6000 applicants were referred to health authorities across the country for post-landing medical surveillance [12]. The majority of referrals were due to tuberculosis and a minority related to positive syphilis or HIV serology [12]. A report by health authorities in Taiwan highlighted that 101,881 foreign migrant workers or 3.7% of all examined over a seven year period had failed the mandatory HA (Table 2) [20]. Failure results in revoking of employment permission and exit from country. In Oman, expatriates developing TB during their stay in the country are deported after conversion to smear-negative, in what is referred to as “the repatriation policy” [7]. Such policies have been viewed by analysts as a possible barrier to early detection and effective treatment of expatriates insofar as it may stigmatize patients and induce them to avoid public health services [21]. HIV and TB control also becomes challenging due to individuals with active disease becoming a “hidden group”, failing to present early to healthcare providers, due to fear of deportation.

**Table 2:** Statistics of failure in the health examination of foreign laborers in Taiwan from 2001 to 2007. Table modified from [20].

Year	HA Type ^1^	Migrants Examined	Number Failed (%)	Parasite (+) ^2^	TB (+) ^3^	HIV (+) ^4^	Syphilis (+)	HBs Ag (+)	Other ^5^
Total for 2007	A	127,121	233 (18%)	88	27	12	9	60	37
	B	342,958	25,649 (7.5%)	25,220	387	13	29	NA	0
Total (2001 to 2007)	A	849,473	2152 (25%)	703	282	112	135	378	542
	B	2,730,708	101,881(3.7%)	98,275	1893	127	284	NA	1300

Notes: **^1^** HA Type A: HA undertaken within three days post-arrival to Taiwan; HA Type B undertaken at 6, 18 and 30 months after entry for work. **^2^** Parasite (+) means the number of people infected with intestinal parasites. **^3^** TB (+) means failure in pulmonary tuberculosis screening. **^4^** HIV (+) means positive antibody reaction to human immunodeficiency virus. HBs Ag (+) means positive reaction to hepatitis B surface antigen. **^5 ^** Other (+) means failure in other items, including positive reactions in pregnancy tests, leprosy tests and urine screenings for narcotics. Urine screening for narcotics was cancelled since January 2004.

## 6. The Need to Link Health Systems with Migration HA Mechanisms at the Country Level

Global public health goods are defined as interventions and services whose benefits cross borders and benefit communities globally [22]. For example, the efforts in controlling TB and HIV provide a public health benefit across borders [23]. HAs provide an opportunity to promote the health of migrants through the initiation of health promotion, disease prevention and curative interventions for conditions that, if left untreated, could have a negative impact on the migrant's health and on the public health of the host community and communities of origin, as well.

A feature of migrant HAs processors is that they often operate within a “vacuum”, with little or no formal linkage to the public health system of the country of origin. We contend that if migrant HA processors are to meaningfully contribute to public health good, then they need to overcome exclusionary approaches, be linked to the national health systems and be complemented by health promotion measures to enhance the health-seeking behavior of migrants. If a prospective migrant is made “non-admissible” at the end of an HA process and is not provided with adequate counseling, treatment and follow-up care, nor contact tracing and preventive care measures are not followed, then we argue that HAs will remain limited as an “immigration functional requirement” of the destination country/foreign employer, rather than providing a public health good. The absence of such public health measures in HA processors may also not take into account international commitments to achieve global health goals as stipulated by the widely adopted World Health Assembly resolution on health of migrants and other such international instruments [24] .

**Figure 2:**
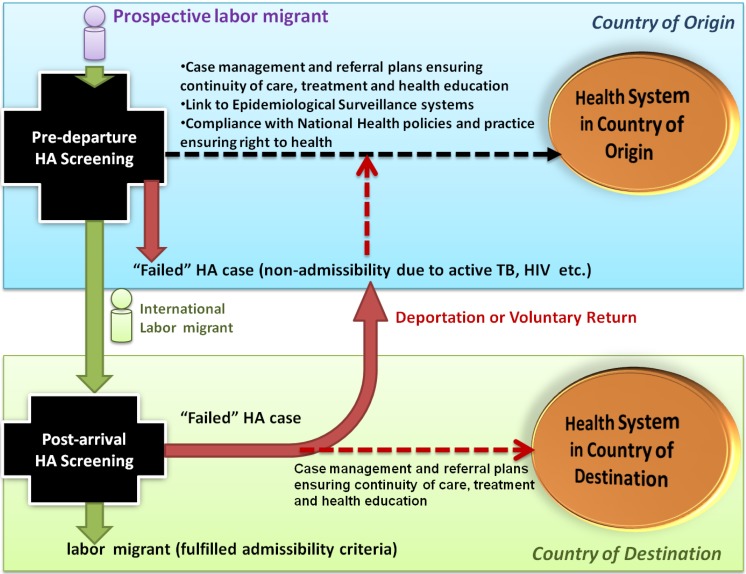
Health assessment model for international labor migrants showing linkages needed to connect HA processors with national health systems in country of origin and country of destination.

Falzon (2012) in the context of exploring the challenge of TB control posits a rhetorical challenge, “Can we turn around the perception embraced by many national public health authorities that “migration is a threat” into an opportunity?” [25]. We argue that if HAs are to adopt more collaborative and meaningful forms of partnership with national health systems, this may indeed lead to greater public health benefits. When suspected cases of HIV and hepatitis C, for instance, are identified as part of the HA process, a case-management plan for the potential migrant may be activated. This may involve the delivery of health education, referral to local health services for treatment and linkage to relevant health promotion programs (Figure 2). Patient consent and participation form a vital part of this follow-up process.

As Figure 2 indicates, ensuring migrants are linked to appropriate medical care irrespective of their HA result, active reporting to national epidemiological surveillance systems and adherence to national health guidelines are examples of adopting a ‘health systems’ approach to migrant HAs. In the case of TB, where strict adherence to strategies of directed observed treatment (DOT) of patients have been identified as critical, the return of migrants affected with TB to home countries during or before the completion of a treatment may contribute to insurgency of drug-resistance. Therefore, better linkage of HA processors with health systems may lead to other benefits, such as ensuring the continuity of the treatment of migrants and curbing the potential spread of drug-resistant TB [26,27].

## 7. Role of Panel Physicians and Immigrant Countries

Engaging destination countries and employment agencies in linking their HA mechanisms to national health systems is also essential in “closing the circle” to enable public health gain. In this regard, the role of immigration country-appointed panel physicians/providers in embracing an enhanced public health agenda needs to be emphasized. It is important to ensure that training and technical instruction (TI) guides for panel physicians formulated by the governments of destination countries emphasize partnerships with national health authorities for disease surveillance requirements (as per the country’s public health regulations) and ensuring treatment and referral plans for those prospective migrants deemed non-admissible based on health status.

A positive development in recent years has been the formation of an Intergovernmental Immigration and Refugee Health Working Group (IIRHWG) formed in 2005 by the governments of the USA, Canada, Australia, U.K. and New Zealand to establish a global panel doctor network. Efforts are being made to strengthen TB diagnostic and screening networks through shared clinics, quality control standards and ensuing policy and practice coherence. Such initiatives may serve to enhance health system linkages and advocacy to improve migrant health and minimize public health security threats.

This group of five countries have also encouraged the establishment in 2009 of an International Panel Physician Association (IPPA) with the mission “to create, maintain and improve a communication network that will enable all participants to establish standardized medical exams based on best practices; give panel physicians, civil surgeons and health experts the ability to share information resources; and promote research and publication on issues related to health and migration”. We underpin the critical role panel physicians can play in leading a possible transformative agenda for immigration HAs. The obligations of recruited screening providers need to be inspired by the same deontological principles of healthcare of the migrants and global health good, stipulated by the inherent relationship between physician and patient. Additionally, more advocacy and new policies are needed *vis-à-vis* migrant recruiters, so as to better realize the these days much emphasized principles of social responsibility for health, also through the use of migrant and employee HAs.

## 8. Conclusions

With declining investments in global public health expenditures, a growing focus on universal health coverage [28], a renewed focus on finding, treating and curing those ‘left behind’ from vertical disease control programs [29] and for promoting active screening for at-risk groups migrants and mobile populations [30], HAs may indeed serve as a global public health good. Despite this potential, HAs remain a largely forgotten “intervention space” in global public health. We argue that the several million HAs performed every year for the scope of migration and international labor offer an important opportunity to enhance universal health coverage.

Discriminatory and excessively exclusionary practices need to be removed as an impediment to patient and global health goals. In countries where a deportation policy is enforced for migrants failing health conditions, the potential to stigmatize vulnerable migrant groups raises ethical and global health concerns. Practices of excludability and forcible return of migrants on medical grounds may contribute to fueling stigma and impeding the recourse to early diagnostics and care. Migrant-sensitive health policies are therefore needed to inform immigration and international recruitment policies [29]. For instance, establishing information systems to evaluate the effectiveness of immigrant screening to allow for evidence-based adjustments of HA policies have been highlighted in the Netherlands [30].

Rather than focusing on the excludability, the HA provides an opportunity to interact with potentially vulnerable migrant groups and to enable health promoting practices. This necessitates strengthening coordination between HA providers and national health systems and a larger partnerships between the public and private actors involved in HA, which leading international health and migration agencies can help build. The public health value of the HA may only be achieved if the HAs move beyond the modus of a mere “disease screening” tool for excludability, to one which ensures adequate quality of care and treatment follow up.
